# A Novel PCR Panel for Bacterial Detection in Lower Respiratory Tract Infections: A Comparative Study with Culture Results

**DOI:** 10.3390/pathogens14101017

**Published:** 2025-10-08

**Authors:** Selda Kömeç, Mehmet Akif Durmuş, Ayşe Nur Ceylan, Ramazan Korkusuz

**Affiliations:** 1 Department of Medical Microbiology, Çam and Sakura City Hospital, Istanbul 34480, Türkiye; drmehmetakifdurmus@gmail.com (M.A.D.); aysenurceylan1011@gmail.com (A.N.C.); 2 Department of Infectious Diseases and Clinical Microbiology, Çam and Sakura City Hospital, Istanbul 34480, Türkiye; ramazankorkusuz@hotmail.com

**Keywords:** quantitative bacterial culture, lower respiratory tract, multiplex PCR

## Abstract

Lower respiratory tract (LRT) infections require rapid and accurate diagnosis. While bacterial culture remains the gold standard, multiplex PCR (mPCR) enables faster and more sensitive detection of multiple pathogens. This study evaluates the Bio-Speedy mPCR panel for 18 bacteria in comparison to conventional culture. A total of 100 LRT samples were analyzed. Complete concordance between the methods was observed in 85% of samples, with mPCR detecting pathogens slightly more frequently (62% vs. 53%). Discrepancies were primarily due to prior antibiotic therapy, low bacterial loads, colonization, or pathogens not included in the PCR panel. The sensitivity and specificity of mPCR were 79.3% and 96.8%, respectively, with negative agreement at 98.9% and positive agreement at 57.0%. Considering culture-negative but clinically relevant PCR-positive results, the sensitivity improved to 98.1% and the positive agreement to 86.7%. mPCR offers early pathogen detection, enabling timely therapy and potentially improving outcomes, particularly in intensive care settings. While culture remains indispensable for viable pathogen identification, combining mPCR with conventional methods provides complementary information, particularly when prior antibiotic use or the presence of fastidious pathogens may compromise culture results. Careful consideration of cost, patient population, and clinical context is recommended for optimal implementation of mPCR panels.

## 1. Introduction

Lower respiratory tract infections (LRTIs) present a variety of symptoms, severity levels, and causative agents. A wide variety of diagnostic methods is essential for detecting and distinguishing the causes of LRTIs. These methods include quantitative bacterial culture, direct staining, and molecular techniques. Cases of pneumonia can be categorized into community-acquired pneumonia (CAP) [1], hospital-acquired pneumonia, ventilator-associated pneumonia, and healthcare-associated pneumonia [2]. Diagnostic and treatment guidelines for CAP are well established, likely due to the relative simplicity of disease presentation and the improved clinical outcomes observed [1]. In contrast, recommendations for hospitalized patients or those having had long-term contact with the healthcare system are more complex. It is hypothesized that prolonged exposure to healthcare settings can lead to colonization and microaspiration of multidrug-resistant organisms, thereby increasing the risk of more severe infections with limited treatment options [3].

According to the WHO’s 2021 report “The Top 10 Causes of Death,” lower respiratory tract infections ranked fifth worldwide, excluding COVID-19. However, in low-income countries, lower respiratory tract infections ranked first [4].

While a Gram stain can give a general indication of a potential infection and the likely causative agent, diagnosing a bacterial infection primarily depends on bacterial cultures. However, the culture process can be cumbersome, especially for critically ill patients, where time is crucial. Multiplex PCR (mPCR) is used as a complementary method to microbiological culture. mPCR provides a faster, more sensitive, and specific method for identifying pathogens in lower respiratory tract infections. Results are available within hours compared to the 24–48 h required for culture results. Despite some limitations associated with mPCR, its ability to quickly identify bacterial agents can be lifesaving. Several studies have demonstrated that the use of mPCR assays reduces the initiation of antibiotics and decreases the duration of antibiotic therapy [5].

mPCR is an advanced molecular biology technique that allows for the simultaneous amplification of multiple target nucleic acid sequences. Using more than one pair of primers in a single reaction enables the concurrent detection of various pathogens or genetic variants [6]. This method is particularly preferred in diagnostic applications due to its time and cost efficiency [7]. mPCR is widely utilized in several fields, including detecting infectious agents, identifying genetic mutations, and differentiating pathogens [8]. It provides rapid and high-throughput analysis by targeting multiple sequences within a single assay [7].

Timely and accurate diagnosis of lower respiratory tract infections is essential for the prompt initiation of effective treatment and to prevent unnecessary antibiotic use [7,9]. The Bio-Speedy^®^ Lower Respiratory Bacteria qPCR Panel (Bioeksen R&D Technologies, Istanbul, Türkiye) was developed to detect 18 bacterial pathogens simultaneously in various sample types, including nasopharyngeal swabs, oropharyngeal swabs, bronchoalveolar lavage, nasopharyngeal aspirate, and sputum samples from individuals suspected of having a lower respiratory infection. This panel utilizes multiplex real-time PCR technology to detect these pathogens at the DNA level by employing target-specific primers and fluorogenic probes tailored to each pathogen. The fluorescent signals produced during amplification allow for real-time detection of the target microorganisms. This approach enables a comprehensive, sensitive, and specific analysis, thereby accelerating the diagnosis process while enhancing accuracy [9].

This study aimed to evaluate the effectiveness of a bacterial lower respiratory tract mPCR panel in comparison to quantitative bacterial culture, which remains the accepted gold standard typically conducted as part of routine diagnostics in microbiology laboratories.

## 2. Materials and Methods

### 2.1. Study Design

The study involved analyzing tracheal aspirate and bronchoalveolar lavage (BAL) specimens collected from adult patients in the intensive care unit (ICU). A total of 100 samples from the lower respiratory tract were examined using both lower respiratory tract (LRT) panel and conventional culture methods. Tracheal aspirates and BAL were analyzed in 75% and 25% of the patients, respectively. In cases of discordant results, where no bacterial growth is detected in the culture but positive results are found in the LRT panel, the use of antibiotics was examined. For these patients with negative culture results or those diagnosed with respiratory tract flora (RTF), and PCR results were positive, gram staining, additional laboratory tests, and imaging were reviewed to determine whether the patient clinically had a lung infection.

### 2.2. Culture Method

For the culture, 10 µL of the sample was inoculated onto 5% sheep blood agar, chocolate agar, and eosin methylene blue agar. A preparation was also made for Gram staining. The inoculated agar plates were incubated in a 3–5% CO_2_ incubator. Bacterial identification was conducted using EXS2600 Matrix-Assisted Laser Desorption ionization-time-of-flight mass Spectrometry (MALDI-TOF MS) (Zybio Inc., Chongqing, China).

A mixture of two or more members of the oropharyngeal microbiota with no predominant pathogens resulted in “Respiratory tract flora bacteria present”.

As the study was prospective, all microorganisms grown in culture were identified, and colony counts were conducted for comparison with those obtained through PCR.

### 2.3. Multiplex PCR Testing

The remaining tracheal aspirate or BAL from the culture was stored frozen at −25 °C until analysis by PCR. The samples were thawed before testing. Tests were performed in a molecular microbiology laboratory within biosafety level 2 (BSL-2) safety cabinets.

Nucleic acid extraction of the specimens was performed using the EXM3000 (Zybio Inc., Chongqing, China) automated extraction system per the manufacturer’s protocol. PCR amplification was performed in accordance with the kit’s thermal cycle protocol, and the manufacturer’s analysis software was used to analyze the fluorescence signal data.

The PCR-based part of the study was performed using the Bio-Speedy^®^ Lower Respiratory Bacteria qPCR Panel kit (Bioeksen R&D Technologies, Istanbul, Türkiye). The pathogens detected by the Bio-Speedy^®^ multiplex PCR kit included *Streptococcus pyogenes*, *Enterobacteriaceae*, *Haemophilus influenzae*, *Mycoplasma pneumoniae*, *Pseudomonas aeruginosa*, *Streptococcus agalactiae*, *Escherichia coli*, *Proteus* spp., *Serratia marcescens*, *Klebsiella pneumoniae*, *Acinetobacter calcoaceticus-baumannii complex*, *Legionella pneumophila*, *Klebsiella aerogenes*, *Enterobacter cloacae complex*, *Streptococcus pneumoniae*, *Staphylococcus aureus*, *Klebsiella oxytoca*, and *Moraxella catarrhalis*.

The results were interpreted qualitatively as either positive or negative, based on the signals from pathogen-specific probes. The results also included outcomes from the internal control targets in the panel to evaluate potential PCR inhibition and assess sample quality. All laboratory tests and evaluations were conducted in accordance with the manufacturer’s technical specifications while minimizing the risk of contamination as much as possible.

## 3. Results

Out of the 100 samples included in the study, 73 were from intensive care units and 27 were from other services. Culture and mPCR assays yielded positive results in 53% and 62%, respectively. Cultures exhibiting bacterial growth not included in the PCR panel were considered negative.

**Figure 1:**
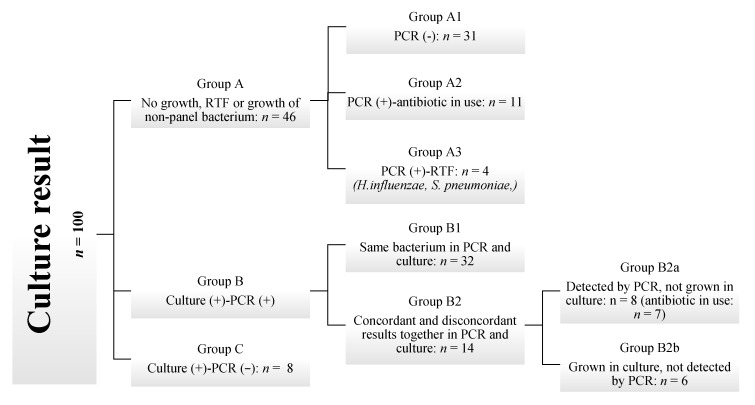
Flowchart of sample categorization by combined culture and PCR findings in suspected lower respiratory tract infections.

Since culture was accepted as the gold standard, the grouping was performed based on culture growth results, and PCR findings were compared accordingly. To facilitate discussion, the culture results were categorized into three main groups: Group A: No growth, RTF, or growth of non-panel bacterium, Group B: Culture Positive-PCR positive, Group C: Culture positive-PCR negative. Subsequently, based on the concordance between PCR and culture results, the findings were classified as completely concordant, partially concordant, or discordant. Group A consists of samples with no growth in culture, growth of RTF, or growth of bacteria not included in the PCR panel (*Candida* spp., *Stenotrophomonas maltophilia*, *Burkholderia cepacia*, *Enterococcus faecalis*, *E. faecium*, *Pseudomonas alcaligenes*). Group A1 consists of samples where the culture was negative or the bacteria present were not included in the PCR panel, and mPCR results were also negative. Group A2 includes samples with no growth in culture but positive mPCR results, taken from patients who had received antibiotics effective against these bacteria. Group A3 contains samples from patients who had not used antibiotics prior to testing and were reported as RTF; still, the mPCR results were positive for H. influenzae and S. pneumoniae, with high CT values. Group B contains samples with positive culture results, in which PCR also yielded a positive outcome. Subgroup B1 refers to cases with positive culture results, where PCR also detected all the bacteria identified by culture. Group B2 includes samples in which, in addition to bacteria common to both methods, there were discrepancies—organisms detected only by PCR or only by culture. Subgroup B2a includes one or more microorganisms detected by PCR but not grown in culture, whereas B2b represents the opposite: at least one bacterium that grew in culture but was not detected by PCR, except for the common microorganisms (Figure 1).

In cases where PCR detected the pathogen but it did not grow in culture, if an antibiotic with potential activity against the PCR-detected organism was administered at the time of sample collection, the lack of growth was considered likely due to antimicrobial suppression. Therefore, such samples were classified as PCR-culture concordant.

When *H. influenzae* and *S. pneumoniae* were detected by PCR, especially at high cycle threshold (Ct) values of >25, and the culture was reported as RTF, the PCR and culture results were considered concordant. Since the upper respiratory flora is present and the number of these bacteria is low, cultures may overlook the growth during evaluation, or it may be considered part of the normal flora.

Group A *(n* = 46), Group B1 (*n* = 32), and seven samples in Group B2a with documented antibiotic use were classified as completely concordant (*n* = 85). One sample in Group B2a without antibiotic use and all samples in Group B2b (*n* = 6) were classified as partially concordant (total *n* = 7). Group C (*n* = 8) was considered discordant. In five of these eight discordant samples, the colony count in culture was <10,000 CFU/mL.

When the microorganism tested positive in both the culture and the PCR, it was categorized as a “true positive” (TP). If the PCR detected the bacteria while the culture was negative, it resulted in a “false positive” (FP). When both tests yielded negative results, it was classified as a “true negative” (TN). Conversely, if the PCR was negative but bacteria grew in the culture, it was labeled a “false negative” (FN). Table 1 presents a cumulative analysis of agreement, sensitivity, and specificity for all analyzed isolates. Table 2 provides a similar analysis specifically for patients undergoing antibiotic treatment. In this latter case, bacteria detected by PCR but not cultured were classified as TP instead of FP.

In 15 samples, bacteria were detected using PCR at high CT values, despite no bacterial growth being observed in cultures or reported as RTF.

Seven types of bacteria were detected, with a colony count exceeding 10,000 CFU/mL in culture: *S. aureus* (n = 4), *K. pneumoniae* (n = 1), *S. marcescens* (n = 1), and *M. catarrhalis* (n = 1), which PCR could not identify.

In 20 samples where PCR detected bacteria but the culture showed growth of less than 10,000 CFU/mL, a review of patient data indicated that 14 (70%) patients were receiving antibiotics that could potentially act against the identified organism.

## 4. Discussion

This study aimed to compare the diagnostic performance of an mPCR panel for detecting bacterial infections in the lower respiratory tract with the standard quantitative bacterial culture method. A total of 100 lower respiratory tract samples were prospectively analyzed using both techniques, with the culture method serving as the gold standard. The findings revealed a high level of concordance between the two methods, with complete concordance observed in 85% of the samples (Figure 1). mPCR is designed to assist in the treatment of severely ill patients, where rapid, targeted therapy is essential. In such cases, the advantages of quick and sensitive detection likely outweigh the risks associated with potential antibiotic overuse. By combining PCR results with other diagnostic data and clinical assessments, physicians can determine the best treatment options more quickly than with conventional methods alone [10].

Time is crucial in treating infections, especially for patients in intensive care units. While the culture method is regarded as the gold standard for diagnosing bacterial infections, it has downsides due to its time-consuming nature and lower sensitivity in patients who are already on antibiotics [11,12]. mPCR provides a significant advantage over traditional culture methods, primarily because it offers rapid results. This quick turnaround is especially beneficial for initiating early treatment in critical patient populations, such as those who are immunosuppressed or have comorbidities [13]. A study on respiratory infections in ICU settings found that mPCR reduced the average hospital stay by two days, demonstrating its potential for conserving resources. Moreover, it improved patient outcomes and decreased antibiotic use by 25% compared to traditional diagnostic methods [9]. Culture-based techniques for microbial identification can limit the diagnosis and management of pneumonia. These methods may fail to detect unculturable, fastidious bacteria such as *Legionella pneumophila* and *Mycoplasma pneumoniae* [14]. In such cases, the use of mPCR techniques can provide significant advantages. However, mPCR faces challenges; high initial costs and the requirement for specialized training can hinder its adoption, particularly in low-resource settings. mPCR results not only improved patient outcomes but also reduced healthcare costs associated with prolonged hospital stays and unnecessary antibiotic use compared to traditional diagnostics [9]. Although the kit costs are higher than conventional methods, its use in selected patient groups (such as those in intensive care, with underlying diseases, or immunosuppressed—where rapid diagnosis and initiation of treatment are critical) is considered acceptable when compared to the total patient costs. In terms of distinguishing between colonization and infection, PCR, particularly at low copy numbers, may be limited in differentiating between these two conditions. Due to the high sensitivity of PCR, false-positive results can occur with low-copy-number DNA samples, which may indicate colonization rather than infection, especially in samples like blood or respiratory secretions, where pathogen presence does not always indicate an active infection [9,15]. To overcome this issue, incorporating additional biomarkers, imaging tests, or quantitative PCR alongside the clinical status of the patients may prove beneficial.

In the study conducted by Milacek et al. [5], cultivation methods and PCR assays yielded positive results in 20% and 24.5% of cases, respectively. Baudel et al. [16] reported a higher pathogen identification rate with mPCR, at 66%, compared to 40% with traditional culture methods. Similarly, Tschiedel et al. [17] found that mPCR detected pathogens more frequently than culture, with rates of 64% and 24%, respectively. In our study, culture methods and mPCR assays produced positive results in 53% and 62% of cases, respectively. We concluded that incorporating the mPCR test improves pathogen detection rates, which can vary based on factors such as the patient population, clinical diagnosis, antibiotic usage, and whether the study is retrospective or prospective.

Milacek et al. [5] found that the concordance rate between culture and PCR for identifying pathogenic microorganisms was 87.7%. In their study, Lee et al. [18] reported a concordance rate of 53.6% for culture-positive results and 86.3% for culture-negative results. Luyt et al. [19] reported a concordance of 73%, with a discordant result of 27%. In Karolyi et al.’s study [20] mPCR and culture yielded non-concordant, partial concordant, and completely concordant results in 13.9%, 30.6%, and 55.6% of the analyzed samples, respectively. Sun et al. [21] found an overall concordance rate of 82.1%. Specifically, the concordance rates were 59.5% for culture-negative PCR-negative, 22.6% for culture-positive and PCR-positive, 4.6% for culture-positive and PCR-negative, and 13.1% for culture-negative and PCR-positive. Our study showed a complete concordance rate of 85%. We observed a partial concordance rate of 7%, while the discordance rate was 8% (Figure 1). Specifically, the concordance rates were 31% for culture-negative PCR-negative, 46% for culture-positive and PCR-positive, 8% for culture-positive and PCR-negative, and 15% for culture-negative and PCR-positive. The rates vary across studies due to differences in study design, interpretation of agreement, sample types, and the PCR assays used.

Studies comparing culture methods with mPCR in respiratory samples have reported varying rates of sensitivity and specificity. Sun et al. [21] found the sensitivity to be 84% and the specificity to be 98%. In contrast, Luyt et al. [19] reported sensitivity and specificity rates of 77.4% and 14.3%, respectively. Gadsby et al. [22] found lower rates, with a sensitivity of 56.9% and a specificity of 58.5%. Meanwhile, Zhuo et al. [23] observed sensitivity and specificity rates of 77% and 94%, respectively. In our study, overall, the sensitivity was found to be 79.31%, while the specificity was 96.84%. The positive agreement rate remained relatively low at 57%, whereas the negative agreement rate was relatively high at 98.86% (see Table 1). This discrepancy may be due to the possibility that PCR missed some culture-positive cases, leading to false negatives, or produced false-positive results in cases of bacterial colonization or antibiotic use. However, when culture-negative but PCR-positive cases were treated as true positives—where the use of antibiotics effective against the bacteria detected by PCR might have suppressed the culture—PCR’s performance significantly improved. In this scenario, the positive agreement rate increased to 86.67%, sensitivity rose to 98.12%, and specificity remained high at 98.84%. This suggests that PCR offers a considerable advantage in detecting pathogens that may not be identifiable through culture, particularly after antibiotic treatment. Research has shown that the use of antibiotics adversely affects bacterial growth in cultures [11,12]. Studies have also shown that mPCR detects more pathogens than culture in patients who have undergone antibiotic treatment [16,17,23]. While the antibiotic may inhibit bacterial growth in culture, it might not be effective in vivo, and the patient’s infection could still persist [24]. In such cases, although culture may not effectively diagnose the infection, PCR’s capacity to address culture negativity resulting from antibiotic use will be crucial for diagnosis and treatment.

A potentially confusing issue arises with the LRT panel’s analytical specificity. Although the panel confirms the presence of microbial DNA, a positive culture result may not always be obtained [3]. This can happen when an antibiotic effective against the bacterium detected by PCR inhibits its growth in culture but does not stop PCR from detecting it [11,12]. This may indicate that residual nucleic acids may still be present following antibiotic therapy. Incorporating additional biomarkers and imaging tests, along with the clinical status of patients, may be beneficial. In our study, PCR results were positive for 15 samples that were either culture-negative or reported as RTF (Groups A2 and A3). Among these patients, 11 had received antibiotics, while in the remaining four, we detected growth of *H. influenzae* and *S. pneumoniae*—organisms that can also be part of the normal flora—at high CT values. The clinical status of these patients was evaluated through Gram staining, additional biomarkers, and imaging. It was found that 10 patients, representing 66.7%, did not have a clinically diagnosed case of pneumonia. This may indicate that mPCR positivity was due to bacterial colonization, the presence of dead bacteria, or residual bacterial DNA.

In the literature, the colony count threshold is generally set at >10,000 CFU/mL, and bacteria that PCR did not detect are highlighted [19,21]. Specifically, among bacteria that mPCR could not detect despite sufficient colony counts, *S. aureus* is noteworthy. The low sensitivity for the detection of *S. aureus* is probably due to its resistance to chemical lysis during DNA extraction [25,26]. To address this issue, Dung et al. [25] and Gadsby et al. [26] indicated in their studies that further research is planned using additional enzymes for the enzymatic lysis of *S. aureus.* In our study, even though the colony count exceeded 10,000 CFU/mL, *S. aureus* (n = 4), *K. pneumoniae* (n = 1), *S. marcescens* (n = 1), and *M. catarrhalis* (n = 1) could not be identified by PCR. Additionally, it is essential to consider that the nature of lower respiratory tract samples (non-homogeneous and mucopurulent) can independently affect both quantitative culture inoculation and the collection of the required sample amount for PCR, which may impact colony counts and CT results. Therefore, cultivation errors or technical challenges related to the extraction process [26] are inevitable. Accurate PCR results depend on both the proper extraction of mucopurulent and nonhomogeneous respiratory tract samples and the specific bacteria being targeted.

A reliable correlation between colony counts in cultures and CT values has not been established [6,17]. As a result, PCR results do not provide information about the bacterial load present in the sample. Detecting bacteria using mPCR methods can sometimes indicate only colonization rather than an active infection, as observed in 4% of our study. We also found no correlation between CT values and colony counts (Pearson r < 0.7). Developing validated quantitative PCR methods could address this limitation. While some researchers aim to achieve quantitative PCR results [25], these methods have not yet been validated in commercially available products. The inability to quantify results is a significant limitation of mPCR. On the other hand, prior exposure to antimicrobial therapy is the most common cause of negative cultures and absent bacterial growth [16,17,19,25] as observed in our study at 19%. Additionally, mPCR was positive in 20 patients, despite colony counts being below 10,000 CFU/mL. Fourteen (70%) of these patients had received antibiotics, suggesting that the low bacterial counts in culture were due to detection by mPCR of dead bacteria or residual DNA with low CT values.

One advantage of traditional culture methods is their ability to detect a wider variety of bacteria compared to mPCR, which has a more limited range of detectable organisms [17,19]. In our analysis, culture identified a pathogen in six cases, despite PCR being negative, because the pathogen was not included in the panel. The limited detection capacity of PCR tests is influenced by the specific test panel used, meaning that some bacteria may not be identified. Adding more bacteria to the panel will increase diagnostic accuracy.

Taken together, combining mPCR-based methods with microbiological culture techniques may improve diagnostic yield.

## 5. Conclusions

With the right indication, traditional culture remains the gold standard for culture-detectable pathogens. However, with appropriate stewardship and interpretation, multiplex panels like LRT can provide valuable clinical information within a critical timeframe. An mPCR assay can offer early pathogen detection in cases requiring rapid diagnosis or treatment, or when pathogens undetectable by conventional culture are suspected.

A limitation of all nucleic acid amplification techniques, including mPCR, is that detection of a pathogen’s DNA/RNA does not necessarily indicate active infection. The microorganism may merely be colonizing, represent a remnant from a previous infection, or represent contamination. In our study, we observed a high correlation between the LRT panel and quantitative bacterial culture.

Therefore, institutions considering a molecular pneumonia panel should perform a careful cost–benefit analysis to determine optimal use for their specific patient population. Protocols may focus testing on patients at the greatest risk for antimicrobial-resistant infections, those currently on antibiotics, and individuals for whom therapy can be tailored based on test results.

## Figures and Tables

**Table 1:** Positive and negative agreement, sensitivity, and specificity in all analyzed isolates.

	No. of Isolates				% Positive Agreement	% Negative Agreement	Sensitivity %	Specificity %
Pathogen	TP	FP	TN	FN				
*Streptococcus pyogenes*	2	3	95	0	40.00	100.00	100.00	96.94
*Haemophilus influenzae*	4	13	82	1	23.53	98.80	80.00	86.32
*Pseudomonas aeruginosa*	11	3	83	3	78.57	96.51	78.57	96.51
*Streptococcus agalactiae*	0	1	99	0	0.00	100.00	0.00	99.00
*Escherichia coli*	4	8	88	0	33.33	100.00	100.00	91.67
*Proteus* spp.	2	0	98	0	100.00	100.00	100.00	100.00
*Serratia marcescens*	4	0	95	1	100.00	98.96	80.00	100.00
*Klebsiella pneumoniae*	19	4	74	3	82.61	96.10	86.36	94.87
*Acinetobacter calcoaceticus-baumannii complex*	6	4	90	0	60.00	100.00	100.00	95.74
*Klebsiella aerogenes*	2	1	97	0	66.67	100.00	100.00	98.98
*Enterobacter cloacae complex*	3	3	93	1	50.00	98.94	75.00	96.88
*Streptococcus pneumoniae*	4	7	89	0	36.36	100.00	100.00	92.71
*Staphylococcus aureus*	5	0	87	8	100.00	91.58	38.46	100.00
*Klebsiella oxytoca*	1	2	97	0	33.33	100.00	100.00	97.98
*Moraxella catarrhalis*	2	2	95	1	50.00	98.96	66.67	97.94
Total	69	51	1362	18	57.50	98.86	79.31	96.84
Total sample n = 100								

**Table 2:** Positive and negative agreement, sensitivity, and specificity in all analyzed isolates, considering PCR-positive results as TP in cases where culture was negative due to prior antibiotic use.

	No. of Isolates				% Positive Agreement	% Negative Agreement	Sensitivity %	Specificity %
Pathogen	TP	FP	TN	FN				
*Streptococcus pyogenes*	4	1	95	0	80.00	100.00	100.00	98.96
*Haemophilus influenzae*	10	7	82	1	58.82	98.80	90.91	92.13
*Pseudomonas aeruginosa*	14	0	83	3	100.00	96.51	82.35	100.00
*Streptococcus agalactiae*	1	0	99	0	100.00	100.00	100.00	100.00
*Escherichia coli*	12	0	88	0	100.00	100.00	100.00	100.00
*Proteus* spp.	2	0	98	0	100.00	100.00	100.00	100.00
*Serratia marcescens*	4	0	95	1	100.00	98.96	80.00	100.00
*Klebsiella pneumoniae*	22	1	74	3	95.65	96.10	88.00	98.67
*Acinetobacter calcoaceticus-baumannii complex*	9	1	90	0	90.00	100.00	100.00	98.90
*Klebsiella aerogenes*	3	0	97	0	100.00	100.00	100.00	100.00
*Enterobacter cloacae complex*	5	1	93	1	83.33	98.94	83.33	98.94
*Streptococcus pneumoniae*	7	4	89	0	63.64	100.00	100.00	95.70
*Staphylococcus aureus*	5	0	87	8	100.00	91.58	38.46	100.00
*Klebsiella oxytoca*	2	1	97	0	66.67	100.00	100.00	98.98
*Moraxella catarrhalis*	4	0	95	1	100.00	98.96	80.00	100.00
Total	104	16	1362	18	86.67	98.70	85.25	98.84
Total sample n = 100								

## Data Availability

Data are available from the authors upon reasonable request.

## Abbreviations

The following abbreviations are used in this manuscript:

LRT	Lower respiratory tract
mPCR	Multiplex PCR
LRTIs	Lower respiratory tract infections
CAP	Community-acquired pneumonia
BAL	Bronchoalveolar lavage
ICU	Intensive care unit
RTF	Respiratory tract flora
MALDI-TOF MS	Matrix-Assisted Laser Desorption Ionization-Time-of-Flight Mass Spectrometry
CT	Cycle threshold
